# Editorial: Revolutionizing immunological disease understanding through single cell multi-omics technologies

**DOI:** 10.3389/fimmu.2025.1628120

**Published:** 2025-05-30

**Authors:** Ying Zhu, Veronica G. Anania, Jennie R. Lill, Zora Modrusan

**Affiliations:** Genentech Inc., South San Francisco, CA, United States

**Keywords:** single-cell omics, single cell RNA sequencing (scRNA-seq), cellular heterogeneity, tumor immunology, spatial technologies, targeted therapies, precision medicine

## Introduction

Single cell approaches have revolutionized our understanding of many different immunological diseases and in this special issue, several new examples are highlighted that pave the way for new treatment options including better understanding of Th17 biology in fibrosis, HIV pathogenesis, IgA Nephropathy pathobiology, and identification of key immune subsets in rheumatoid arthritis and other autoimmune diseases. Spatial technologies have furthered our knowledge of gene expression and protein colocalization, opening new paths for studying tissue-specific cellular dynamics. Advancements like the SENSE method for cryopreserving whole blood have simplified single-cell analyses, making them more viable for clinical use. Innovative multiplexing strategies and developments in proteomics and Raman spectroscopy are broadening the capabilities of single-cell technologies, allowing for comprehensive profiling that can enhance precision medicine. Together, these advances highlight the potential of single-cell omics to lead the future of immunological research and clinical practice, facilitating the creation of novel therapeutic strategies and personalized medicine.

## Understanding disease mechanisms through single-cell RNA sequencing

Deng et al. studied fibrotic skin diseases like keloids, hypertrophic scars, and scleroderma, characterized by excessive fibroblast growth and extracellular matrix buildup. They used fluorescence-activated cell sorting to isolate CD45+ immune cells from keloid and normal scar tissues, then applied scRNA-seq to map the immune cell landscape. The study found a significant increase in Th17 cells, which promote fibroblast proliferation, collagen expression, and migration via IL-17A secretion. This increase in Th17 cells in other fibrotic conditions suggests a common mechanism in skin fibrosis, advancing the understanding of these diseases and identifying potential therapeutic targets.

Hong et al. used multiplex cytokine assays and scRNA-seq to explore immunological differences between ACPA-positive and ACPA-negative early rheumatoid arthritis (eRA) patients. They found that ACPA+ eRA patients had higher levels of interferon-gamma (IFN-γ) and interleukin-12 (IL-12), indicating a Th1 immune response. The study identified 17 distinct cell types, with notable expansions of IL1B+ proinflammatory monocytes, CD8+ CCL4+ T cells, and IL7R+ T cells in ACPA+ eRA. These cells showed upregulated IFN-γ response genes, suggesting enhanced IFN-driven monocyte-T cell interactions. IFITM2 and IFITM3 were identified as potential biomarkers for ACPA+ eRA. These findings indicate that ACPA+ eRA is characterized by a more active IFN-mediated immune response, potentially guiding personalized treatment strategies targeting type I and II interferon pathways.

scRNA-seq has been crucial in understanding immunity related to pathogenic invasion. Knoll et al. conducted a comprehensive study on immune cell reprogramming in people living with HIV (PLHIV), revealing persistent functional changes in monocytes even with long-term antiretroviral therapy (ART). Using various omics technologies, the study identified significant transcriptomic changes in monocytes, indicating an “anti-viral” state with upregulated IFN signaling pathways, like acute HIV infection. This suggests ongoing immune activation despite ART. The research also explored drug repurposing to reverse the pro-inflammatory monocyte phenotype in PLHIV, identifying sunitinib and doxycycline as promising candidates. These findings support the larger 2000-HIV multicenter cohort study, highlighting the need for transcriptomics to define disease endotypes and predict outcomes. The study emphasizes verifying chromatin-level differences via ATAC-seq and further exploring monocyte-mediated immune dysregulation. Despite a small sample size, it lays the groundwork for future research to refine therapeutic strategies and understand the immune landscape in PLHIV under ART.

Nephrotic syndrome (NS) is a severe form of IgA nephropathy (NS-IgAN) with unclear pathogenesis, marked by immune cell imbalances and kidney damage. Chen et al. used scRNA-seq on peripheral blood mononuclear cells and kidney cells from pediatric NS-IgAN patients to investigate this condition. They found increased intermediate monocytes (IMs) expressing VSIG4, MHC class II molecules, and genes related to oxidative phosphorylation. Classical and non-classical monocytes showed elevated CCR2, possibly linked to kidney injury. Two regulatory T cell subsets were identified, with Treg2 cells expressing high CCR4 and GATA3, potentially aiding kidney recovery. Podocyte injury was associated with increased CCL2, PRSS23, and epithelial-mesenchymal transition genes. PTGDS was suggested as a potential podocyte marker due to its decreased expression after injury. This study provides insights into NS-IgAN pathogenesis and could guide future targeted therapies.

Huang et al. describe the integration of single-cell and spatial transcriptomic analyses to unravel the cellular heterogeneity and molecular mechanisms underlying ulcerative colitis (UC), a chronic inflammatory bowel disease characterized by immune dysregulation. By identifying distinct monocyte subtypes and leveraging machine learning techniques, two key genes, GNG5 and TIMP1, were highlighted as central to UC pathogenesis. GNG5, downregulated in UC, is implicated in anti-inflammatory pathways such as PPAR signaling, while TIMP1, upregulated, exhibits pro-inflammatory effects and correlates with T cell exhaustion markers like TIGIT and CTLA4. Spatial transcriptomic data, immunohistochemical validation in human UC lesions, and experimental findings from a DSS-induced colitis mouse model confirmed these gene expression patterns. TIMP1 was further shown to co-localize with macrophages and promote Th17-driven inflammation, suggesting its dual role in macrophage activation and immune depletion. These findings provide a foundation for developing targeted therapeutic strategies aimed at mitigating chronic inflammation and immune dysfunction in UC.

## Spatial technologies

Spatial technologies are essential for pinpointing specific cell types and gene expression locations. Moos et al. demonstrated how spatial-temporal single-cell transcriptomic sequencing can analyze genetic mutations in pulmonary epithelial nodes related to pulmonary fibrosis (PF) and interstitial lung diseases. Using a clinical PF dataset and a murine model with SP-C gene mutations, they investigated monocyte/macrophage changes in fibrotic lungs. The study found heterogeneous activation of CD68+ macrophages, especially near injury sites. Ingenuity Pathway Analysis showed asynchronous activation of extracellular matrix reorganization and ApoE signaling in alveolar macrophages. scRNA-seq identified pro-fibrogenic signaling from Trem2+ macrophages. Although genetic deletion of ApoE had limited impact on inflammation, the study suggests ApoE as a biomarker for active macrophages in tissue remodeling. These findings provide insights into macrophage heterogeneity and cell-cell interactions in fibrotic diseases.

Advances in spatial proteomics and protein colocalization are crucial for understanding cellular mechanisms and developing novel algorithms. Rhomberg-Kauert et al. introduced Molecular Pixelation (MPX), a method that provides spatial information on surface proteins in single cells, allowing for *in silico* graph representation of protein neighborhoods. To analyze this data modality, local assortative methods were adapted to assess spatial relationships, enabling evaluation of pairwise colocalization and similarity among multiple proteins. MPX was tested on datasets showing its ability to detect stimuli effects, such as T cells treated with a chemokine to study uropod formation, and cancerous B-cell lines treated with rituximab, providing insights into cell polarity. This computational approach enhances understanding of immune responses and cell surface protein reorganization, potentially guiding new therapeutic designs. MPX offers high throughput, sensitivity, and three-dimensional analysis, surpassing traditional microscopy, and enabling deep phenotyping at single-cell resolution. The method can also analyze other biological spatial data represented as graphs, showcasing its broad applicability and potential to advance spatial proteomics.

Sample preparation is often a bottleneck in single-cell methodologies, especially with biofluids. Satpathy et al. developed the SENSE method for single-step cryopreservation of whole blood (WB), streamlining cell suspension preparation for scRNA-seq. This method overcomes the limitations of labor-intensive multistep processes unsuitable for clinical use. In a comparative analysis of six blood samples, the SENSE method produced highly viable single-cell suspensions, with 22,353 cells showing a viability rate of 86.3 ± 1.51%. It yielded high-quality transcriptomic profiles comparable to traditional PBMC methods and showed higher T-cell enrichment, allowing for detailed T-cell subtype characterization. Both methods captured transcriptional and cellular networks across cell types, with minimal batch effects, except in myeloid cells. The SENSE method’s simplicity and effectiveness make it promising for widespread clinical and research adoption, facilitating single-cell assays and translational research.

Khoshbakht et al. introduced a label-free sample multiplexing strategy based on the souporcell algorithm, enabling cost-effective scRNA-seq and flow cytometry analyses of paired blood and skin samples. This protocol addresses the complexity and cost of current methods, applicable to both healthy and inflamed skin. It allows simultaneous RNA and protein analysis on the same lesion, reducing costs by 2–4 times. The strategy minimizes batch effects and examines the impact of different enzymatic incubation durations (1, 3, and 16 hours, with and without enzyme P) on flow cytometry results. It includes bioinformatic demultiplexing and a step-by-step guide, making it accessible for newcomers. This approach aims to enhance single-cell analysis accessibility, potentially extending to other dermatological disorders and aiding in understanding immune mechanisms and identifying new therapeutic targets.

Single-cell omics techniques for clinical samples have traditionally focused on genomic, transcriptomic, and more recently, proteomic methodologies. Raman spectroscopy has emerged as a complementary bioanalytical tool due to its ability to characterize the biophysical properties of biomolecules. Chadokiya et al. review how molecular tumor characterization is crucial for identifying predictive biomarkers to improve precision immunotherapy. However, challenges like tumor heterogeneity and limited biomarker efficacy hinder accurate treatment predictions. This study highlights label-free Raman spectroscopy as a non-invasive tool for profiling precision immunotherapy, capable of unifying various omics data. With its ability to distinguish immune cell types and detect molecular changes, Raman spectroscopy offers a promising approach for enhancing treatment prediction and monitoring in cancer care. As it evolves, Raman spectroscopy could become a cost-effective, patient-focused tool integrated into clinical practice for precise immunotherapy.

## Future directions

Future developments in single-cell omics technologies are set to transform our understanding and treatment of immunological and tumor-related diseases. Integrating multi-omics data including genomic, transcriptomic, proteomic, and metabolomic at the single-cell level, along with spatial information, will provide a comprehensive view of cellular states and interactions, aiding in the discovery of new biomarkers and therapeutic targets. Advances in artificial intelligence and machine learning will be vital for analyzing the large datasets from these technologies, enabling predictive models for disease progression and treatment response. Enhancing spatial omics technologies will offer insights into cell and molecular organization within tissues, enriching our understanding of tissue architecture and function. Expanding single-cell techniques to less-studied cell types and rare diseases will uncover new areas of human health. Making single-cell omics cost-effective and user-friendly will be crucial for their integration into routine clinical practice, allowing personalized healthcare for more patients. These advancements will deepen our understanding of complex biological systems and lead to innovative therapies and precision medicine tailored to individual needs.

